# The Identification and Characterization of the *KNOX* Gene Family as an Active Regulator of Leaf Development in *Trifolium repens*

**DOI:** 10.3390/genes13101778

**Published:** 2022-10-01

**Authors:** Jinwan Fan, Gang Nie, Jieyu Ma, Ruchang Hu, Jie He, Feifei Wu, Zhongfu Yang, Sainan Ma, Xin Zhang, Xinquan Zhang

**Affiliations:** Department of Forage Breeding and Cultivation, College of Grassland Science and Technology, Sichuan Agricultural University, Chengdu 611130, China

**Keywords:** *Trifolium repens*, *KNOX* gene family, genome-wide, leaf development, expression analysis

## Abstract

Leaves are the primary and critical feed for herbivores. They directly determine the yield and quality of legume forage. *Trifolium repens* (*T. repens*) is an indispensable legume species, widely cultivated in temperate pastures due to its nutritional value and nitrogen fixation. Although the leaves of *T. repens* are typical trifoliate, they have unusual patterns to adapt to herbivore feeding. The number of leaflets in *T. repens* affects its production and utilization. The *KNOX* gene family encodes transcriptional regulators that are vital in regulating and developing leaves. Identification and characterization of *TrKNOX* gene family as an active regulator of leaf development in *T. repens* were studied. A total of 21 *TrKNOX* genes were identified from the T. repens genome database and classified into three subgroups (Class I, Class II, and Class M) based on phylogenetic analysis. Nineteen of the genes identified had four conserved domains, except for *KNOX5* and *KNOX9*, which belong to Class M. Varying expression levels of TrKNOX genes were observed at different developmental stages and complexities of leaves. *KNOX9* was observed to upregulate the leaf complexity of T. repens. Research on *TrKNOX* genes could be novel and further assist in exploring their functions and cultivating high-quality *T. repens* varieties.

## 1. Introduction

Leaves are the leading sites and essential organs of plants in photosynthesis, and directly affect morphogenesis and sugar accumulation in plants [1]. For forage, leaves are the primary and critical animal feed, which determine the biomass yield and quality to a great extent [2,3]. *T. repens* is an indispensable legume species, widely cultivated in temperate pastures owing to its nutritional value and ability to fix atmospheric nitrogen. [4]. Although the leaves of *T. repens* are typical trifoliate, they have unusual patterns to adapt to herbivore feeding [5]. The number of leaflets in *T. repens* affects its production and utilization. 

Homeobox genes are central genes that regulate the development of higher organisms [6], one of which is the *Knotted1-like homeobox* (*KNOX*) gene family [7]. The transcriptional regulators encoded by the *KNOX* gene family serve a variety of purposes in plant physiology and development, especially leaf development [8,9]. Based on sequence similarity, gene structure, phylogeny, and expression levels, the *KNOX* gene family is divided into three subclasses: Class I *KNOX* (*KNOXI*), Class II *KNOX* (*KNOXII*), and Class M *KNOX* [10].

*KNOXI* genes have shown expression pattern diversity in species with simple and compound leaves. In simple leaf species, such as *Arabidopsis thaliana*, *KNOXI* genes were continuously observed to be down-regulated after expression in leaf primordia [11]. The *KNOXI* genes were reactivated during early primordia development in most plant species (*Solanum lycopersicum*, and *Cardamine hirsuta*) with compound leaves [8,12]. Nevertheless, a permanent downregulation of *KNOXI* genes was observed at the incipient sites of leaf primordia formation in *Medicago truncatula* and *Pisum sativum*, which belong to the large inverted repeat-lacking clade (IRLC) of papilionoid legumes [13,14,15], indicating that compound leaf development in IRLC species may not be mediated by *KNOXI* genes. Fascinatingly, the leaf complexity of *M. truncatula* was increased by ectopic *STM*/*BP*-like *KNOXI* (*KNOX1*, *KNOX2*, *KNOX6*) activity [15].

The functions of Class II *KNOX* genes are widespread in comparison to Class I [10,16,17]. *Knotted Arabidopsis Thaliana 3* (*KNAT3*) and *KNAT7* were found to participate in seed coat mucilage biosynthesis redundantly in *A. thaliana* [18]. *KNAT4* controlled seed physical dormancy [19], whereas *KNAT7* exhibited a vital role in secondary cell wall synthesis [20]. Additionally, CIN-TCP transcription factors and KNOXII formed a robust differentiation module. It suppressed the KNOXI-CUC network and leaflet initiation, thus promoting simple leaf architecture in *A. thaliana* [21]. 

*KNATM* is a member of Class M in *A. thaliana*, located at the boundaries of mature and proximal–lateral regions of primordia organs. It has been inferred that *KNATM* plays an influential role in leaf proximal–distal patterning [10]. *Fused Compound Leaf1* (*FCL1*) is a member of Class M *KNOX* genes that was found to play a positive role in boundary separation and proximal–distal axis development of compound leaves in *M. truncatula* [22]. This suggests that the Class M *KNOX* genes effectively enhance the development of compound leaves in *M. truncatula*.

*T. repens* is a legume species belonging to IRLC with trifoliolate compound leaves [23]; thus, the effects of *KNOX* genes on *T. repens* leaf development may be equally complex and variable. In the current research, the genes of the *TrKNOX* family were identified and synthetically analyzed in *T. repens*, along with their structure, chromosomal location, evolutionary relationships, and classification of *TrKNOX*. Finally, the expression patterns of *TrKNOX* genes were screened from different leaf developmental stages and various genotypes in *T. repens*.

## 2. Materials and Methods

### 2.1. Identification of TrKNOX Genes in T. repens

The genome resource information of *T. repens* was gathered from a previous study [24]. The query sequences were generated using the amino acid sequences of the 9 *KNOX* genes from *A. thaliana* using the TAIR (https://www.arabidopsis.org/, accessed on 2 June 2021) and BLAST programs (TBLASTN and BLASTP) on the Phytozome (http://www.phytozome.net/, accessed on 2 June 2021) [25,26]. *TrKNOX* genes in *T. repens* were identified by two BLAST methods and similar redundant genes were eliminated simultaneously. The *KNOX* gene family was screened for conserved structural domains predicted by the NCBI Batch CD-search tool (https://structure.ncbi.nlm.nih.gov/Structure/burps/bwrpsb.cgi, accessed on 3 June 2021) in *T. repens* [27]. Protein sequences of TrKNOX were used to investigate the protein length, molecular weight, theoretical pI, and subcellular location using the ExPasy website (https://www.expasy.org/, accessed on 3 June 2021) and softberry (http://www.softberry.com/, accessed on 3 June 2021) [28]. Analysis of cis-acting elements was based on the online tool PlantCARE (http://bioinformatics.psb.ugent.be/webtools/plantcare/html/, accessed on 5 June 2021) and TBtools software (https://github.com/CJ-Chen/Tbtools/, accessed on 5 June 2021) used to predict and visualize the results [29].

### 2.2. Phylogeny and Classification of TrKNOX Genes 

Protein sequences of the KNOX family transcription factor in *A. thaliana* were obtained using iTAK (http://itak.feilab.net/cgi-bin/itak/db_browse.cgi, accessed on 10 June 2021) [30]. The phylogeny of the TrKNOX proteins was investigated in comparison to *A. thaliana* sequences. Based on the neighbor-joining (NJ) method, gaps were deleted and at 1000 bootstraps support, the phylogenetic trees of the *TrKNOX* gene family were constructed on MEGA version 7.0 [31].

### 2.3. Gene Structure, Motif, and Chromosomal Localization Analysis of TrKNOX Genes

Jalview software (http://www.Jalview.org/, accessed on 12 June 2021) was used to investigate the structures of *TrKNOX* genes. Additionally, Tbtools was used to analyze the constituents of exons and introns of *TrKNOX* genes [29]. The motif of TrKNOX proteins was analyzed on MEME (https://meme-suite.org/meme/tools/meme, accessed on 12 June 2021), with the following parameters: motif width 6-200 and number set to 10.We used TBtools to locate *TrKNOX* genes on *T. repens* chromosomes [29].

### 2.4. Evolutionary Analysis of TrKNOX Genes with Several Related Species

The protein sequences of the KNOX family transcription factor in *M. truncatula* and *T. pratense* were obtained using iTAK website (http://itak.feilab.net/cgi-bin/itak/db_browse.cgi, accessed on 15 June 2021) [30]. The phylogeny of the TrKNOX proteins were investigated using *M. truncatula* and *T. pratense* sequences. Based on the NJ method, gaps were deleted and at 1000 bootstraps support, the phylogenetic trees of the *TrKNOX* gene family were constructed on MEGA version 7.0 [31].

### 2.5. Protein–Protein Interaction (PPI) Network Construction of the Crucial TrKNOX Genes in T. repens Using the Interolog Approach

Generally, the interolog approach is based on the inference of protein–protein interaction (PPI) information accumulated from other organisms [32]. In the current study, inferences were based on PPI information from three plant species (*A. thaliana*, *M. truncatula*, and *T. pratense*). Based on STRING 11.5 (https://string-db.org, accessed on 15 May 2022), protein interaction information was obtained for the three temperate plants. Additionally, *T. repens* PPI networks were visualized using Cytoscape (https://cytoscape.org, accessed on 17 May 2022) [33]. The Jalview software (http://www.jalview.org/, accessed on 18 May 2022) was used to investigate the differences in amino acid sites of TrKNOX proteins and their homologous protein in *A. thaliana*, *M. truncatula*, and *T. pratense*. 

### 2.6. Plant Materials

In expression pattern analysis of *TrKNOX* genes during leaf development, *T. repens*. cv. Haifa, a widely used commercial variety, was propagated in pots measuring: diameter 16.5 cm, height 10 cm, and base 12 cm, employing asexual propagation from cuttings, in a greenhouse for 14 h daylight at 22 °C and for 10 h darkness at 20 °C. The leaves were thereafter collected from five representative developmental stages: the period when the stem wraps the young leaves (T_1_); the period when the young leaves are just protruding from the stem (T_2_); the period of complete overlap of leaflets (T_3_); the period of 50 percent leaflet expansion (T_4_); and mature leaf (T_5_). The collected samples were frozen in liquid nitrogen for approximately 15 min and thereafter stored at −80 °C until use.

Two pairs of genotypes (Haifa and its variant singleton Tr-171 with a four-leaf phenotype (46%); Tr-007 and its filial generation HybC1-132 with a phenotype of 3–7 leaflets) were used to analyze the expression patterns of *TrKNOX* genes in *T. repens* with different leaf types. The four genotypes were cultured from cuttings under the same conditions as previously described. The leaves were thereafter collected at T1 stages. The collected samples were frozen in liquid nitrogen for approximately 15 min and thereafter stored at −80 °C until use.

### 2.7. Expression Patterns Analysis of TrKNOX Genes Using qRT-PCR

The expression level analysis of *TrKNOX* genes used qRT-PCR to investigate the expression levels of *TrKNOX* genes. The primers used were designed using Primer Premier 5 (Table S1). A stably expressed *Trβ-Actin* gene was used as the internal control for *T. repens* (Table S1) [34]. *TrKNOX9* was used as the control gene in five different leaf developmental stages. The qRT-PCR reaction was performed in triplicates with SYBR Premix Ex Taq II and thereafter, the data were analyzed using the 2^−(ΔΔCt)^ method [35]. Origin 2022 was used to analyze the data.

## 3. Results

### 3.1. Identification of TrKNOX Genes in T. repens 

A total of 21 *TrKNOX* genes were identified from the *T. repens* genome database. The sequence bit scores from high to low with the KNOX domain HMM profile and the *TrKNOX* genes were abbreviated as *TrKNOX1* to *TrKNOX21*. In the current study, the basic information of 21 *TrKNOX* family genes was analyzed (Table 1). The results obtained showed that the longest protein was TrKNOX7 (437 aa), while the smallest protein was TrKNOX5 (134 aa); the MWs of proteins ranged from 14,759.7 Da (TrKNOX5) to 48822.03 Da (TrKNOX7). The pI ranged from 4.61 (TrKNOX19) to 8.97 (TrKNOX12), with those of 90.5% being less than 7, meaning that most TrKNOX proteins are acidic amino acids. In the predicted subcellular location, the information obtained shows that 20 TrKNOXs were located in the nucleus and only one was extracellular (TrKNOX19). The basic information of the *TrKNOX* gene family varied widely, indicating there are various functions performed by these genes.

Analysis of cis-acting elements of 21 *TrKNOX* genes revealed that a great number contained abundant cis-acting elements corresponding to light response and hormone response (gibberellin and auxin induction, respectively), and many cis-acting elements were related to stress resistance (drought and wound-responsive) (Figure S1; Table S2).

### 3.2. Phylogenetic Analysis and Classification of TrKNOX Genes

**Figure 1:**
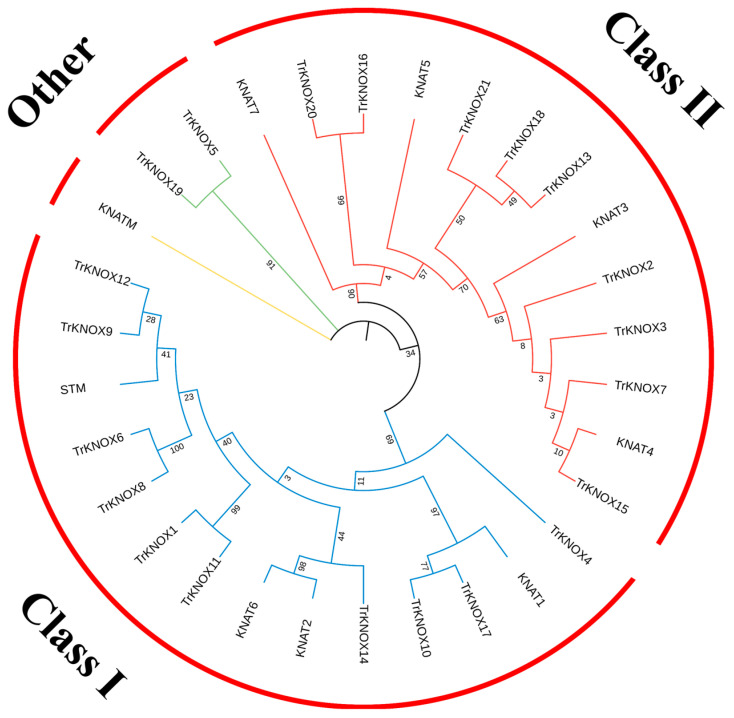
An unrooted phylogenetic tree showing relationships between the KNOX family proteins in *T. repens* and *A. thaliana*. The tree divided the TrKNOX proteins into three subclasses. Neighbor-joining (NJ) was used to derive the phylogenetic trees.

An unrooted phylogenetic tree was constructed based on the sequences of KNOX proteins of *A. thaliana* and *T. repens* and used to explore their evolutionary relationships. Based on their homology to KNOX proteins in *A. thaliana*, 21 TrKNOX proteins were divided into three subgroups (Figure 1). Class I and Class II had 10 and 9 members, respectively. However, TrKNOX5 and TrKNOX19 did not fall into either category. According to the current results, it could be assumed that *TrKNOX* family gene functions are multi-faceted in *T. repens*, and the findings were observed to be consistent with previous studies conducted in *A. thaliana*. 

### 3.3. Gene Structure and Conserved Motifs Analysis of TrKNOX 

**Figure 2:**
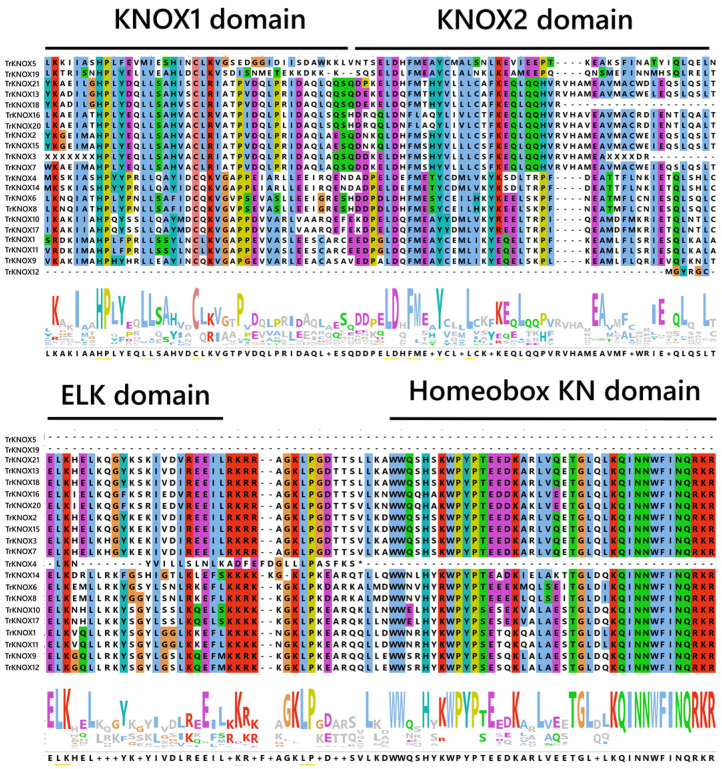
Sequence alignment and logo of KNOX domain from Trifolium repens. KNOX1 domain, KNOX2 domain, ELK domain, and Homeobox KN domain have been marked.

The sequence alignment was performed using the full-length protein sequences of TrKNOX. It showed that most of the KNOX family proteins had four domains: the KNOX1 domain, the KNOX2 domain, the ELK domain, and the Homeobox KN domain. Interestingly, TrKNOX5 and TrKNOX19 contained only two conserved structural domains, KNOX1 and KNOX2 (Figure 2). 

**Figure 3:**
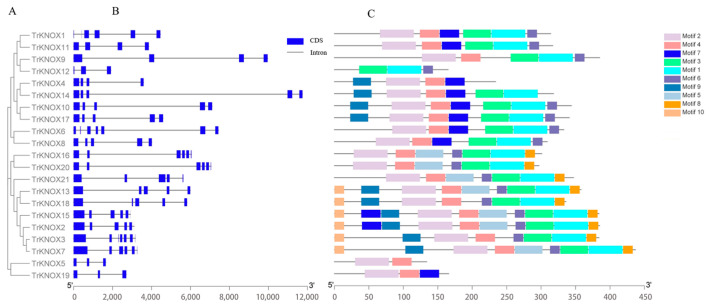
Gene structure and architecture of conserved protein motifs for the *TrKNOX* genes of Trifolium repens based on their phylogenetic relationships. (**A**) The phylogenetic tree was constructed based on the CDS sequences of *T. repens* KNOX family proteins by the MEGA 7 software. (**B**) CDS-intron structure of *T. repens KNOX* genes. Blue boxes indicate exons; black lines indicate introns. (**C**) The motif composition of TrKNOX proteins. The motifs, numbered 1–10, are displayed in different colored boxes. The sequence information for each motif is provided in Table S3. The length of the TrKNOX protein can be estimated using the scale at the bottom.

The DNA sequences of *TrKNOX* genes were analyzed and their structures compared (Figure 3B). Ten conserved motifs were identified based on the MEME analysis (Figure 3C); the amino acid sequence for each motif is listed in the supplementary data (Table S3). It was observed that these conserved motifs vary in length from 15 to 50 amino acids, and motifs 1, 2, 3, and 4 were the most conservative in structure. TrKNOX2 and 15 had the most abundant motifs comprising of 10 types, whereas, TrKNOX5 had two types (motif 2 and 4). TrKNOX proteins in the third subclass were found to lack most of the motifs, whereas the proteins in the first subclass had more of motif 5 and 8 in comparison to the second subclass (Figure 3C). The findings suggests that the functions of the three subclasses could be diverse.

### 3.4. Chromosomal Locations and Evolutionary Analysis of TrKNOX Genes with Several Related Species 

**Figure 4:**
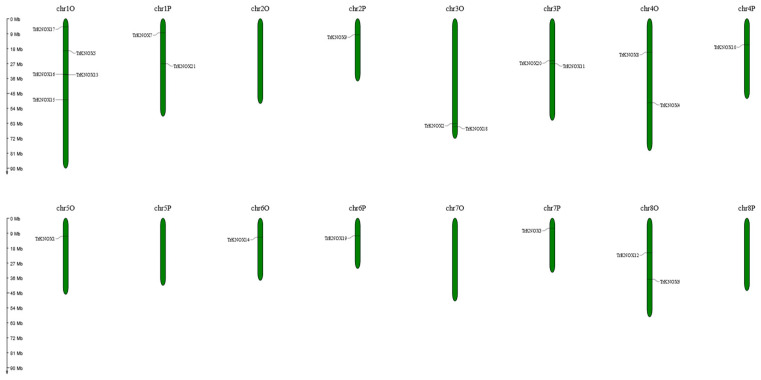
Distribution of *TrKNOX* genes among 16 chromosomes in *T. repens*. Vertical lines represent the chromosomes of *T. repens*. The chromosome number is on each chromosome. The scale on the left represents chromosome length.

There were 21 *TrKNOX* genes distributed unevenly on 16 chromosomes of *T. repens* in total (Figure 4). Chromosome 1O had the most significant number of genes (5, ~23.81%), followed by chromosomes 3O, 3P, 4O, and 8O (2, ~9.52%), respectively. Chromosomes 2P, 4P, 5O, 6O, 6P, and 7P had one gene only (1, ~4.76%), and chromosomes 2O, 5P, 7O, 8P, had no gene distribution. Most of the *TrKNOX* genes were located in the middle and upper part of the chromosomes, with two genes (*TrKNOX2* and *TrKNOX18*) located at the bottom of chromosome 3O.

**Figure 5:**
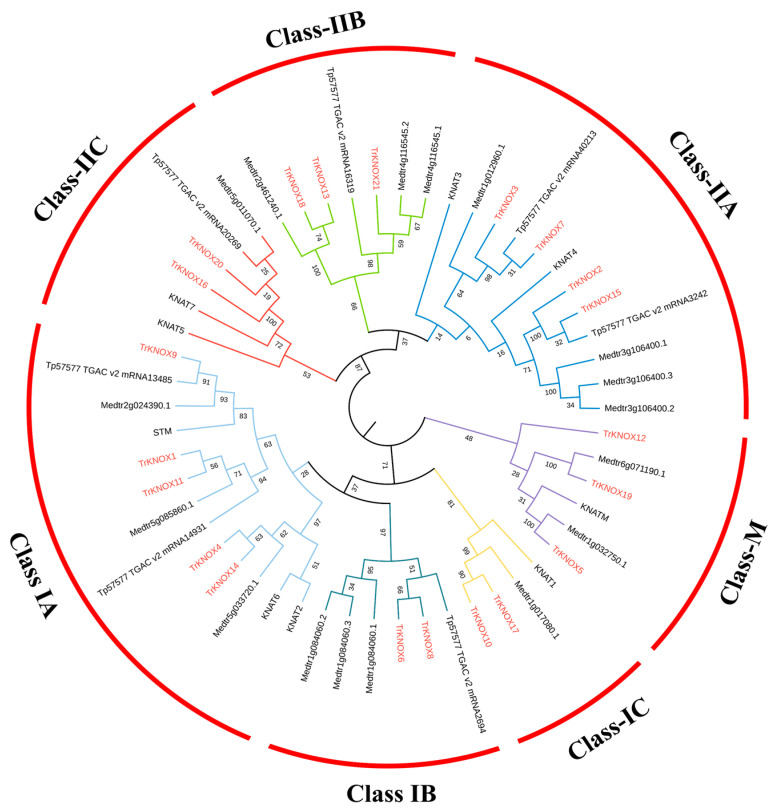
Phylogenetic relationships and motif compositions of KNOX family proteins of four related species. The tree divided the TrKNOX proteins into three subgroups, Class I, Class II, and Class M.

To study the diversity of *TrKNOX* genes evolution and to predict their functions, a phylogenetic tree was constructed using protein sequences of *T. repens*, *A. thaliana*, *M. truncatula*, and *T. pratense* (Figure 5). The constructed phylogenetic tree showed that, all *TrKNOX* genes were placed into separate clades of three subclasses. Classes I and II were further placed into three clades of subclasses: IA, IB, and IC, and IIA, IIB, and IIC, respectively.

### 3.5. Expression Analysis of TrKNOX Genes during Leaf Development 

**Figure 6:**
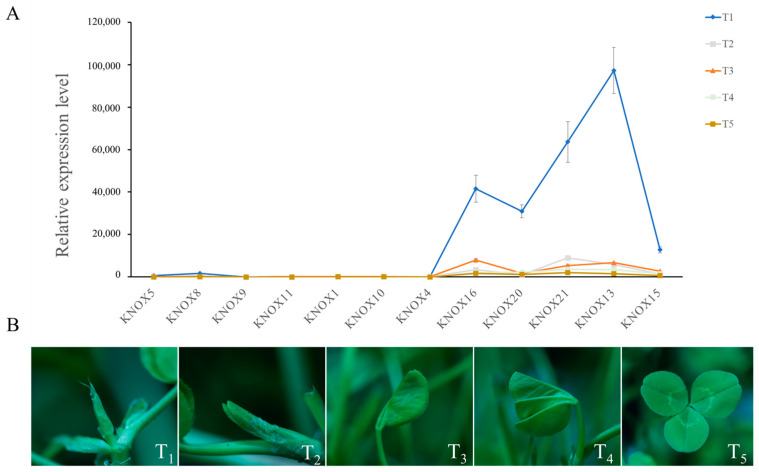
Gene expression of 12 *TrKNOX* genes during leaf development in *T. repens*. (**A**) The relative expression patterns of 12 *T. repens KNOX* genes were investigated at five leaf development stages through qRT-PCR method. Error bars were obtained from three measurements. Small letter(s) above the bars indicate significant differences (α = 0.05, LSD) among the treatments. (**B**) Leaf morphology of *T. repens* at five leaf development stages.

*KNOX* gene family has been studied concerning plant development in many plants, especially in leaf development. To explore the role of the *TrKNOX* genes in *T. repens* leaf development, 12 genes were selected randomly and investigated using RT-qPCR among branches (Figure 5). The expression levels of 12 *TrKNOX* genes across the five stages of *T. repens* leaf development were evaluated (Figure 6).

At the T_1_ stage, 12 selected genes showed the highest expression levels. The genes in Class II (*TrKNOX16*, *20*, *21*, *13*, and *15*), were highly expressed in comparison to the genes in Class I and Class M. *KNOX13*, *KNOX21*, and *KNOX16* exhibited the highest expression levels from the T_1_ to T_3_ stages. Additionally, the expression level of *TrKNOX21* was the highest at T_4_ and T_5,_ whereas at T_1_ and T_3_, the expression level of *TrKNOX9* was noted to be the lowest. It was also observed that the expression level of *TrKNOX11* was the lowest at other stages and was barely expressed at T_5_. The current study showed that the highly expressed genes were all from Class II, and the least expression was noted in Class I genes. It could be deduced that the expression levels of all selected genes decreased with leaf development (Figure 6); thus indicating that the *TrKNOX* gene family has a possible novel function at the T_1_ stage of *T. repens* leaf development. 

### 3.6. The Correlation of Expression for 21 TrKNOX Genes of T. repens

**Figure 7:**
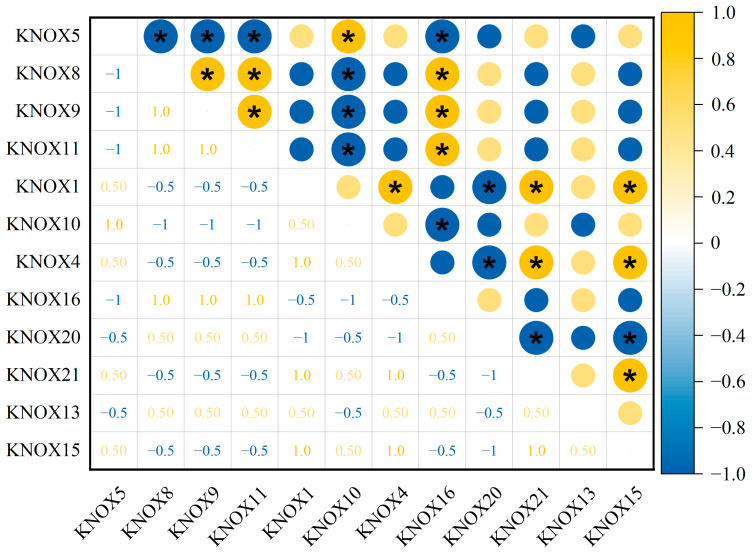
The correlations between the TrKNOX gene relative expression. Yellow: positively correlated; blue: negatively correlated. The marks of * indicate significant differences (*p* ≤ 0.05, Spearman) among the treatments.

As could be seen from the results in Figure 6, the *TrKNOX* gene family played a critical role at the T_1_ stage in leaf development of *T. repens*. The relative expression profiles of 21 *TrKNOX* genes were studied at T_1_ (Figure 7) and the findings showed that the expression of *TrKNOX15* (a member of Class II) was significantly negatively correlated with the expression of the members of Class I (*KNOX1* and *KNOX4*). Accordingly, the expression of *TrKNOX21* (a member of Class II) was significantly negatively correlated with the expression of *KNOX4*, a member of Class I (Figure 7). These results suggested that some genes of Class II may inhibit the expression of some genes of Class I at T_1_ of *T. repens* leaf development.

### 3.7. Expression Patterns of TrKNOX Genes during Leaf Development in T. repens with Different Leaf Types

**Figure 8:**
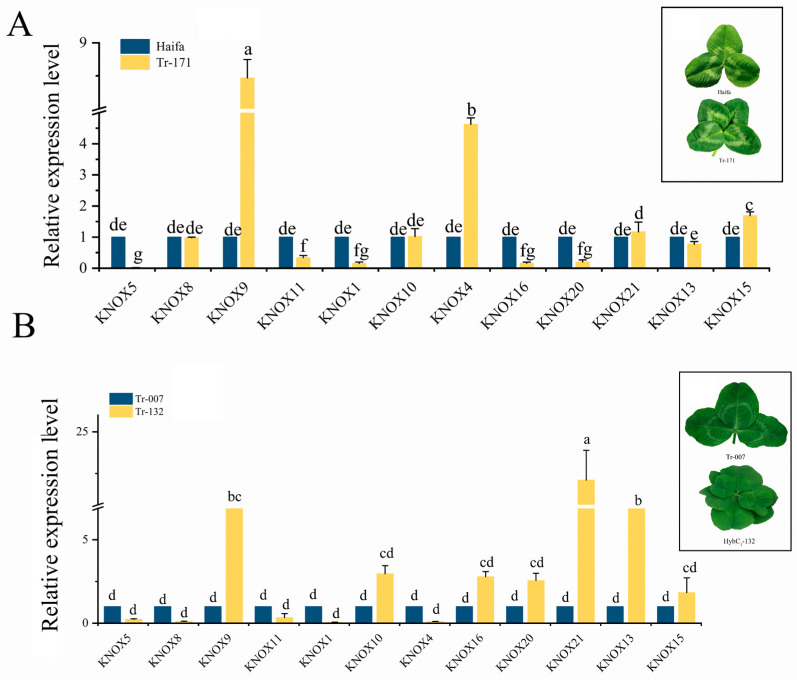
The relative expression of 12 *TrKNOX* genes of leaf development in *T. repens* with different leaf types. (**A**) 12 gene expression patterns of the *TrKNOX* family in Haifa and Tr-171. (**B**) 12 gene expression patterns of the *TrKNOX* family in Tr-007 and HybC1-132. Error bars were obtained from three measurements. Each value is the mean ± SD of three biological replicates; different letters above bars indicate statistically significant difference at *p* < 0.05.

The expression pattern of *KNOX* genes in the early stage of leaf development affects the leaf types of mature leaves. RT-qPCR was used to investigate the roles of *TrKNOX* genes in *T. repens* of different leaf types (Figure 8).

Tr-171, a variant singleton of Haifa with a four-leaf phenotype (46%), showed that, the expression levels of *TrKNOX* genes in Tr-171 were not similar to those in Haifa (Figure 8A). The expression of *TrKNOX9*, *TrKNOX4,* and *TrKNOX15* in Tr-171 was observed to be significantly higher in comparison to that of Haifa. Additionally, the expression levels of *TrKNOX5*, *TrKNOX11*, *TrKNOX1*, *TrKNOX16,* and *TrKNOX20* in Tr-171 were markedly lower in comparison to those in Haifa. The observed results showed that *TrKNOX9*, *TrKNOX4,* and *TrKNOX15* may have positive significance for the number of compound leaves in *T. repens*; *TrKNOX5*, *TrKNOX11*, *TrKNOX1*, *TrKNOX16* and *TrKNOX20* may inhibit leaflet emergence of *T. repens*.

HybC1-132, a F_1_ generation of Tr-007 with phenotype of 3–7 leaflets, was explored and its regulation mechanism of leaf development between HybC1-132 and its parent Tr-007 was studied. Further, the expression patterns of *TrKNOX* genes were compared (Figure 8B); the findings showed that the expression of *TrKNOX9*, *TrKNOX21*, and *TrKNOX13* in HybC1-132 were significantly higher in comparison to those of Tr-007. Based on the results obtained, it could be assumed that *KNOX5*, *KNOX9*, and *KNOX21*, which belong to Class M, Class I, and Class II, respectively, may be fundamental in the leaf development of *T. repens*.

### 3.8. Protein–Protein Interaction (PPI) Network Prediction of Three TrKNOX Proteins

**Figure 9:**
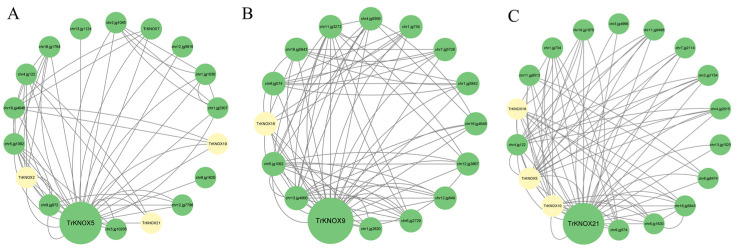
Interactions between three essential proteins (**A**. TrKNOX5, **B**. TrKNOX9, and **C**. TrKNOX21) regulate leaf development and other proteins in *T. repens*. Nodes represent protein, and yellow indicates other TrKNOX protein family members; green represents other proteins.

The interaction between proteins is a direct phenomenon that is involved in biological signal transmission, gene expression regulation, and other vital processes. In the current study, an interolog-based method was utilized to construct PPI networks of TrKNOX5, TrKNOX9, and TrKNOX21 (Figure 9). Additionally, the interaction information of each protein in PPI is shown in the supplementary data (Tables S5–S7). The results showed that the PPI network of TrKNOX5 consists of 58 interactions linked to 18 proteins (Figure 9A). In the network, chr2.jg1045 (a member of the protein kinase superfamily) and chr16.jg4646 (a member of the BLH protein family) were identified, and both were found to be involved in the regulation, growth, and development of plants. TrKNOX2, TrKNOX10, and TrKNOX21 were observed to also exist in the interaction network of TrKNOX5. Additionally, chr12.jg7796, a homologous protein of PALM1, and chr9.jg972, a homologous protein of LFY, play a vital role in compound leaf development in legumes [36].

It could be noted that TrKNOX9 PPI network consists of 77 interactions linked to 16 proteins: (Figure 9B), chr12.jg3867, a member of the GRAS protein family, and chr6.jg574, chr16.jg4646, and chr16.jg5843, members of the BLH protein family in the network. TrKNOX16 was also observed to exist in the interaction network of TrKNOX9. Additionally, chr11.jg3272 and chr6.jg2729 are homologous proteins of CUC2 and CUC1, and chr13.jg4900, a homologous protein of AS1, has a vital role in leaf development.

The PPI network of TrKNOX21 consists of 78 interactions linked to 18 proteins (Figure 9C). In the network, chr6.jg574 and chr8.jg1620, members of the BLH protein family, were identified, and TrKNOX5, TrKNOX10, and TrKNOX16 were found to also exist in the interaction network of TrKNOX21.

In addition, the protein sequences of TrKNOX5, TrKNOX9, and TrKNOX21 were compared with their homologous proteins in *A. thaliana*, *M. truncatula*, and *T. pratense,* respectively (Figure S2). There were some amino acid sites variations in the KNOX1 and KNOX2 domains of KNOX5, KNOX9, and their homologous proteins, which may be one of the significant reason for the functional differences of leaf development.

## 4. Discussion

The *KNOX* gene family codes for transcriptional regulators, which are common in plants and have various functions in plant development. Currently, nine genes in *A. thaliana* [7,10], 12 in *M. truncatula* [15,36], 13 in *Oryza sativa*, and 15 in *Populous tirchocarpa* have been identified [37]. In the current study, we identified 21 *TrKNOX* genes in *T. repens*, and the number of *TrKNOX* genes was found to be more abundant in comparison to most species.

The structures of Class M members differ from those of Class I and Class II in *T. repens*. TrKNOX transcription factors, domains, and motifs were found be related to DNA binding, transcriptional activity, and protein interaction [38]. The *KNOX* family genes are divided into Class I, Class II, and Class M. The M class members of the *KNOX* gene family contain only two conserved structural domains: KNOX1 and KNOX2. In the current study, TrKNOX5 and TrKNOX19, which belong to Class M, contained only KNOX1 and KNOX2 domains, respectively. The length of proteins in Class M members was shorter in comparison to those of other classifications. In KNOX proteins, the lengths ranged from 300–400 aa in *A. thaliana*, whereas in KNATM (a member of Class M), the length was found to be 142 aa [7,10]. The length of FCL1 (Medtr6g071190.1), a representative member of Class M in the KNOX family proteins of *M. truncatula*, was found to be 161 aa, [36]. In the research, the lengths of TrKNOX proteins ranged from 300–400 aa; TrKNOX5 and TrKNOX19 were 134 aa and 166 aa, respectively. The results could be attributed to the fact that the functions of Class M genes differ from those of Class I and II, thus reflecting the diversity of *TrKNOX* genes and their functions.

The expression patterns of *TrKNOXI* genes might be similar to those of genes in *P. sativum* and *M. truncatula*. In simple-leaved species (*A. thaliana* and *Tobacco*), *KNOXI* genes are expressed in the shoot apical meristem (SAM) and are down-regulated after leaf initiation [21,39]. As for most compound-leaved species (*S. lycopersicum* and *C. hirsuta*), the expression of *KNOXI* was found to be reactivated in leaflet primordia [21,40,41]. However, the legume species belonging to the IRLC (*P. sativum*, *M. truncatula*) were similar to those of simple-leaved species; *KNOXI* genes were found to be permanently down-regulated in the incipient sites of leaf primordia initiation [13,40]. *T. repens* is a compound-leafed legume belonging to the IRLC. In this study, the expression levels of *TrKNOXI* genes decreased significantly after the T_1_ stage during leaf development in *T. repens*.

KNOXII may inhibit the KNOXI-CUC network at the early stage of leaf development in *T. repens*. Previous studies showed that the genes from Class II suppressed the KNOXI-CUC network and leaflet initiation with CIN-TCP transcription factors in *A. thaliana* [21]. In the present study, the expression of *TrKNOX15* (a member of Class II) was significantly negatively correlated with the expression of Class I members (*KNOX1* and *KNOX4*). Additionally, the expression of *TrKNOX21* (a member of Class II) was also found to be significantly negatively correlated with the expression of *KNOX4*. On the other hand, KNOX9 interacted with chr11.jg3272 and chr6.jg2729 (which are homologous proteins of CUC2 and CUC1). It could be deduced from the current research that there might be an inhibitory relationship between KNOXII and KNOXI-CUC in *T. repens*. However, the inhibitory mode of the CIN-TCP-KNOXII complex on the KNOXI-CUC network exists in *T. repens*; further research needs to be conducted to ascertain the findings.

*TrKNOX9* are involved in regulating the complexity of leaves in *T. repens*. Previous studies showed that the STM acts broadly on leaf complexity in *A. thaliana*. Ectopic expression of STM during leaf development increased leaf complexity [9,42]. In *M. truncatula*, the leaves in *knox1* mutant were found to be normal, in comparison to the leaf complexity increasing with ectopic *MtKNOX1* activity in *M. truncatula* [15]. The current study exhibited that, at the early stage of leaf development in Tr-171 and Haifa, the changes in *TrKNOX9* and *TrKNOX4* expression were the highest. *TrKNOX9* and *TrKNOX4* belong to Class I and *TrKNOX9* is in the same branch as *STM* and *MtKNOX1* (*Medtr2g024390.1*) in the evolutionary tree, indicating that *TrKNOX9* is the homologous gene of *STM* and *MtKNOX1*. This perhaps could mean that the function of *TrKNOX9* is similar to that of *STM* and *MtKNOX1*, promoting the complexity of leaves in *T. repens*. The expression levels of *TrKNOX9*, *TrKNOX21*, and *TrKNOX13* in HybC1-132 were significantly higher in comparison to those of Tr-007. The results further suggests that *TrKNOX9* might promote the formation of leaflets, thus increasing the complexity of leaves in *T. repens*.

Perhaps a strong reciprocal relationship between KNOX and BLH exists in *T. repens*. Currently, studies have reported the interactions between KNOX and BLH, which serve a crucial function in the regulation of plant growth and development on land [43,44,45]. Recently, a putative interaction of KNOX with BLH was shown to regulate leaf and fruit development in tomatoes [46]. In the current study, all three PPI networks contain the interaction between KNOX and BLH; thus, there could be a strong reciprocal relationship between the two proteins in *T. repens*. Several proteins that might interact with TrKNOX proteins were predicted, thereby providing helpful information for future studies on the functional identification of *KNOX* genes in *T. repens* and other related species.

## 5. Conclusions

In the current study, the gene structure, chromosome localization, sequence homology, and expression pattern of the *TrKNOX* gene family in *T. repens* were comprehensively analyzed for the first time. Additionally, expression analysis of *TrKNOX* genes in developmental leaves in *T. repens* showed that the known and new *TrKNOX* genes, especially *TrKNOX9*, were crucial in regulating leaf development in *T. repens*. Although further studies are needed to elucidate their functions, genome-wide analysis of *TrKNOX* in *T. repens* will be of importance in discovering possible new *TrKNOX* genes and, thus, could act as the basis for the functional verification and identification of *TrKNOX* genes in the future.

## Figures and Tables

**Table 1:** List of the 21 TrKNOX genes identified in the study.

Gene	Gene ID	Protein Length	Mw (KDa)	pI	Localization
*TrKNOX1*	chr5.jg1646.t1	314	36,092.01	8.35	Nuclear
*TrKNOX2*	chr3.jg10199.t1	385	43,542.46	5.74	Nuclear
*TrKNOX3*	chr15.jg975.t1	384	-	-	Nuclear
*TrKNOX4*	chr4.jg7618.t1	234	25,798.44	4.67	Nuclear
*TrKNOX5*	chr1.jg2841.t1	134	14,759.70	4.83	Nuclear
*TrKNOX6*	chr8.jg5547.t1	333	37,894.84	5.86	Nuclear
*TrKNOX7*	chr9.jg1297.t1	437	48,822.03	5.64	Nuclear
*TrKNOX8*	chr4.jg2912.t1	309	35,077.32	5.21	Nuclear
*TrKNOX9*	chr10.jg1505.t1	385	43,606.47	6.46	Nuclear
*TrKNOX10*	chr12.jg2462.t1	344	39,142.8	6.32	Nuclear
*TrKNOX11*	chr11.jg4376.t1	317	36,226.06	6.38	Nuclear
*TrKNOX12*	chr8.jg3024.t1	165	19,418.92	8.97	Nuclear
*TrKNOX13*	chr1.jg5055.t1	358	40,653.28	5.97	Nuclear
*TrKNOX14*	chr6.jg1763.t1	318	36,055.49	5.16	Nuclear
*TrKNOX15*	chr1.jg7372.t1	384	43,455.34	5.74	Nuclear
*TrKNOX16*	chr1.jg5003.t1	301	34,059.36	6.46	Nuclear
*TrKNOX17*	chr1.jg696.t1	341	38,931.62	6.28	Nuclear
*TrKNOX18*	chr3.jg10469.t1	336	-	-	Nuclear
*TrKNOX19*	chr14.jg1682.t1	166	19,106.12	4.61	Extracellular
*TrKNOX20*	chr11.jg4105.t1	297	33,554.85	6.31	Nuclear
*TrKNOX21*	chr9.jg4282.t1	347	39,827.41	5.69	Nuclear

## Data Availability

All data generated or analyzed during this study are included in this article and its attached documents. The *T. repens* genome resources were downloaded from the EMBL/GenBank data libraries (Bioproject PRJNA523044, https://www.ncbi.nlm.nih.gov/genbank/, accessed on 2 June 2021). The protein sequences of the KNOX transcription factor gene in *A. thaliana*, *M. truncatula*, and *Trifolium pratense* were downloaded from the iTAK website (http://itak.feilab.net/cgi-bin/itak/db_browse.cgi, accessed on 10 June 2021).

## Supplementary Materials

The following supporting information can be downloaded at: https://www.mdpi.com/article/10.3390/genes13101778/s1, Figure S1: Analysis of 1500 bp cis-acting element upstream of *TrKNOX* genes. Blocks of different colors represent different action elements.; Table S1: RT-qPCR primers in the study; Table S2. Cis-acting elements on the promoters of *TrKNOX* genes in *T. repens*; Table S3. Analysis of conserved motifs of TrKNOX protein in *T. repens*; Table S4. Specific location information of *TrKNOX* genes on chromosome; Table S5. The interaction information of the PPI network for TrKNOX5 protein; Table S6. The interaction information of the PPI network for TrKNOX9 protein; Table S7. The interaction information of the PPI network for TrKNOX21 protein.

## Abbreviations

*KNOX*	*Knotted1-like homeobox*
IRLC	Inverted repeat-lacking clade
*STM*	*SHOOTMERISTEMLESS*
*BP*	*BREVIPEDICELLUS*
*KNAT*	*Knotted Arabidopsis Thaliana*
*FCL1*	*Fused Compound Leaf1*
MW	Molecular weight
PI	Isoelectric point
NJ	Neighbor-joining
PPI	Protein–protein interaction
