# Supplementary material for: The Identification and Characterization of the KNOX Gene Family as an Active Regulator of Leaf Development in Trifolium repens
Source: Genes (Basel). 2022 Oct 1;13(10):1778. doi: 10.3390/genes13101778 (PMC9601826; doi:10.3390/genes13101778)
Supplement: Supplementary file 1 [file genes-13-01778-s001.zip › figues S1-S2.pptx]

## Slide 1
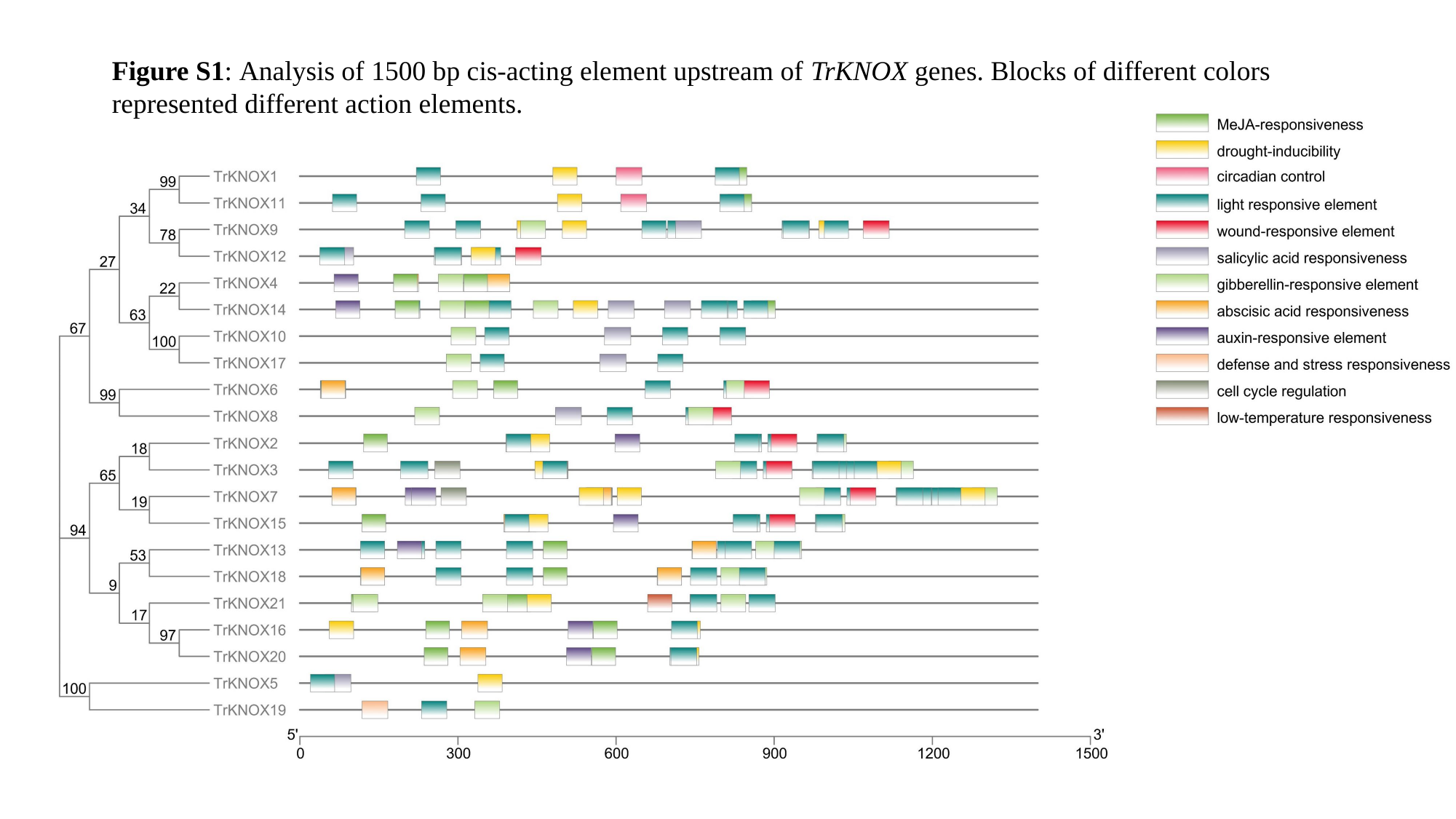

Figure S1: Analysis of 1500 bp cis-acting element upstream of TrKNOX genes. Blocks of different colors represented different action elements.

## Slide 2
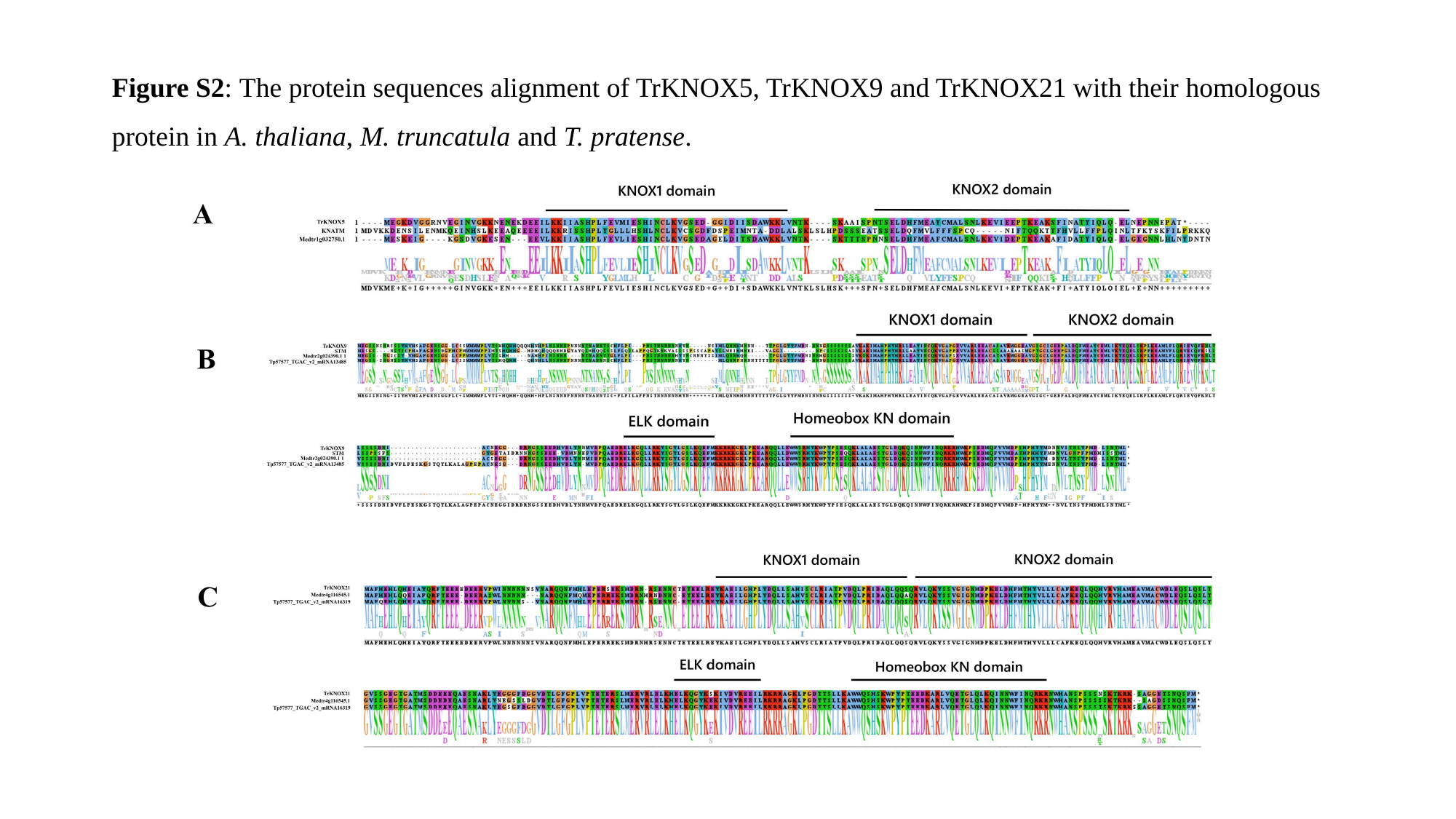

Figure S2: The protein sequences alignment of TrKNOX5, TrKNOX9 and TrKNOX21 with their homologous protein in A. thaliana, M. truncatula and T. pratense.
